# A high throughput screening system for studying the effects of applied mechanical forces on reprogramming factor expression

**DOI:** 10.1038/s41598-020-72158-5

**Published:** 2020-09-22

**Authors:** Jason Lee, Miguel Armenta Ochoa, Pablo Maceda, Eun Yoon, Lara Samarneh, Mitchell Wong, Aaron B. Baker

**Affiliations:** 1 Department of Biomedical Engineering, University of Texas at Austin, 1 University Station, BME 5.202D, C0800, Austin, TX 78712 USA; 2 Institute for Cellular and Molecular Biology, University of Texas at Austin, Austin, TX USA; 3 The Institute for Computational Engineering and Sciences, University of Texas at Austin, Austin, TX USA; 4 Institute for Biomaterials, Drug Delivery and Regenerative Medicine, University of Texas at Austin, Austin, TX USA

**Keywords:** Biological techniques, Biophysics, Drug discovery, Stem cells

## Abstract

Mechanical forces are important in the regulation of physiological homeostasis and the development of disease. The application of mechanical forces to cultured cells is often performed using specialized systems that lack the flexibility and throughput of other biological techniques. In this study, we developed a high throughput platform for applying complex dynamic mechanical forces to cultured cells. We validated the system for its ability to accurately apply parallel mechanical stretch in a 96 well plate format in 576 well simultaneously. Using this system, we screened for optimized conditions to stimulate increases in Oct-4 and other transcription factor expression in mouse fibroblasts. Using high throughput mechanobiological screening assays, we identified small molecules that can synergistically enhance the increase in reprograming-related gene expression in mouse fibroblasts when combined with mechanical loading. Taken together, our findings demonstrate a new powerful tool for investigating the mechanobiological mechanisms of disease and performing drug screening in the presence of applied mechanical load.

## Introduction

Cellular reprograming is a process in which differentiated, mature cells are converted into induced pluripotent stem cells (iPSCs) through the expression of specific transcription factors. Generation of iPSCs is typically achieved through the expression of octamer-binding transcription factor-4 (Oct-4), Kruppel-like factor 4 (Klf-4), Sex Determining Region Y Box 2 (Sox2), and v-Myc Myelocytomatosis Viral Oncogene Homolog (c-Myc)^1^. The discovery of the means to reprogram mature cells to iPSCs and subsequently differentiate them into defined lineages has immense potential to revolutionize cell-based therapeutics, drug screening and scientific investigation of many diseases. However, current protocols for creating iPSCs are often inefficient and time-consuming^2,3^. Moreover, genetic modification of cells to overexpress the transcription factors has the risk of creating mutations in the reprogrammed cells and tumorigenicity is a major concern with iPSC-based therapies^4^. Several groups have found that small molecules can replace some or all of the transcription factors that induce cellular reprograming^5–8^, supporting that reprogramming is possible in the absence of exogenous gene delivery.

Biophysical forces are receiving increased recognition as important modulators of biological processes in many fields including cancer^9,10^, stem cell biology^11–14^ and embryological development^15^. Recent work has suggested a link between the mechanical environment and pluripotency^16^. In addition, mechanical force can lead to chromatin remodeling and altered binding of transcription factors^17,18^. Recent studies have also shown that application of mechanical strain can reduce the expression of pluripotency in mouse embryonic stem cells^19,20^. In human pluripotent stem cells (hPSCs), mechanical strain helped to maintain pluripotency through increased expression Nodal, transforming growth factor-β (TGF-β), and Activin^21,22^. However, in other studies, mechanical strain reduced the expression of pluripotency transcription factors and signaling pathways related to pluripotency in iPSCs^23^. Mechanical stretch also enhanced the reprograming of cells treated with retrovirus-delivered pluripotency transcription factors without altering the efficiency of viral transduction^24^. Other studies have linked alterations in pluripotency to changes in cell shape or substrate stiffness^25–27^. Thus, while the mechanical environment has a powerful effect on cellular reprogramming it is unclear how best to optimize applied forces and/or mechanical environment to enhance pluripotency.

In this study we have developed a flexible, high throughput system for the studying cellular mechanobiology. Systems for studying applied mechanical forces to cultured cells often use flexible cell culture surfaces that can be expanded through applied mechanical forces^28–30^, expansion of the membrane can be induced through the displacement of a piston^31,32^, pneumatic suction^33^ or direct traction on the membrane^34^. These systems have been reviewed extensively in several past and recent reviews^28,29,35^. Systems using a rotational motor with a cam to drive piston motion are limited in that they can apply only one waveform without modifying the cam shape^31^. Pneumatic systems are commercially available but also have limitations in the dynamics of the pneumatic system and ability to apply only a single strain at one time^36^. The throughput for these systems is often a major limitation, which prevents the performance of screening studies for pathway and drug discovery. While there have been studies to expand the throughput of mechanical loading system for cells, these systems often make compromises in the uniformity of the strains applied, dynamics of strain application and robustness in order to achieve increased throughput^37,38^. Here, we developed a high throughput system that allows the application of mechanical stretch to cells in a high throughput format of up to 576 wells simultaneously. The system is configured with removable multi-well plates that can be used in high content imaging systems and plate reading assays. In addition, the system allows the application of arbitrary waveforms with highly controlled dynamics, allowing the simulation of complex physiological forces.

Using this mechanobiological screening platform, we examined whether there were mechanical conditions that could induce somatic cell (fibroblasts) to express reprogramming factors that are involved in the development of pluripotency. We found that high level of mechanical strain and physiological loading induced the expression of reprograming factors in mouse embryonic fibroblasts (MEFs). By combining mechanical conditioning with a drug screen of small molecule signaling modulators, we also identified compounds that could further enhance the expression of Oct-4 in mouse fibroblasts. Our studies reveal that optimized mechanical stimulation combined with small molecule inhibitors can markedly enhance the expression of reprogramming factors in fibroblasts in the absence of transgene delivery.

## Results

### High throughput system for studying stem cell mechanobiology

**Figure 1:**
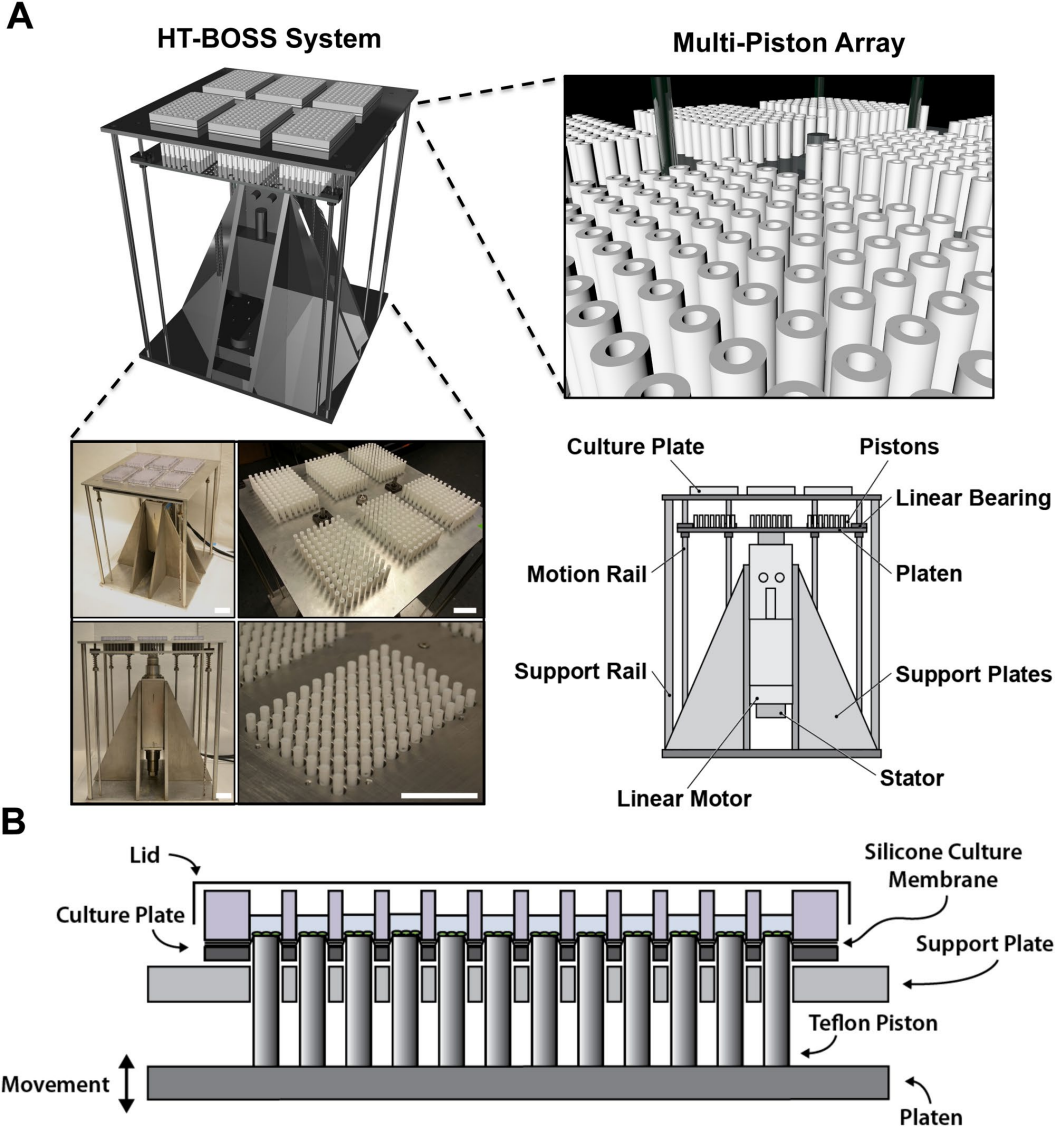
High throughput system for applying mechanical forces to cultured cells. (**A**) The system applies mechanical strain to cells cultured in a 96 well plate format. A platen with 576 pistons is moved by a linear motor to displace a flexible membrane in six 96-well plates with flexible culture surfaces. Bar = 5 cm. (**B**) The culture plate consists of two plates that connect to hold a thin silicone membrane that serves as a culture surface. Thicker silicone membranes serve to seal each well and prevent leakage between the wells. Teflon pistons mounted on the platen are displaced into the membrane to apply mechanical strain to the cells cultured in each well.

**Figure 2:**
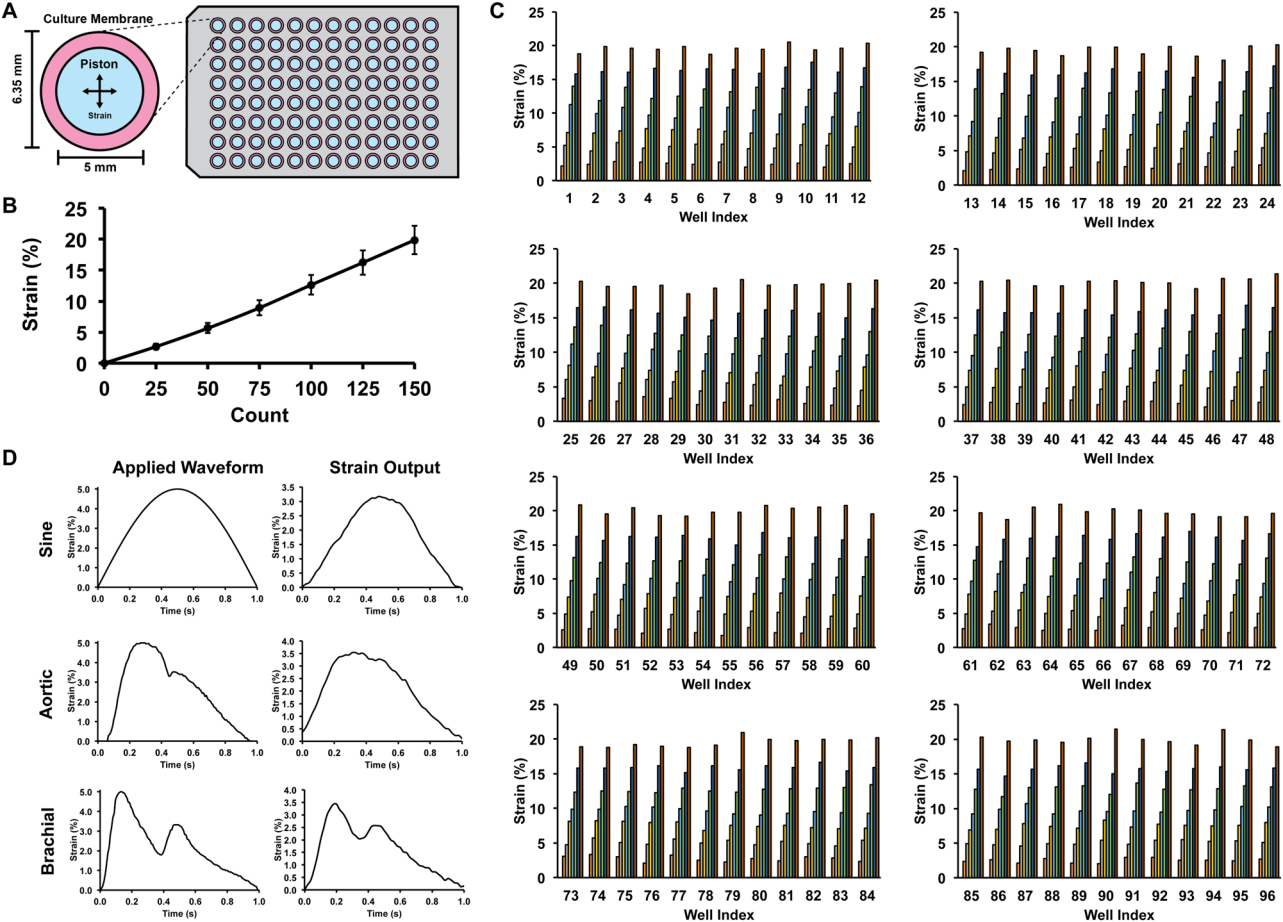
Calibration of the mechanical applied by the high throughput system and validation of mechanical strain waveform dynamics. (**A**) Top view of the system showing the relative geometry of the piston and the culture surface. (**B**) Average strain applied by the system with vertical displacement of the platen. Each motor count is 10 µm of vertical displacement. (**C**) Well-by-well measurement of strain for a 96 well plate under load application. (**D**) Dynamic strain waveforms produced by the system through control of the platen motion with the linear motor. The aortic and brachial waveforms simulate the strain on the arterial wall during the cardiac cycle in the body.

**Figure 3:**
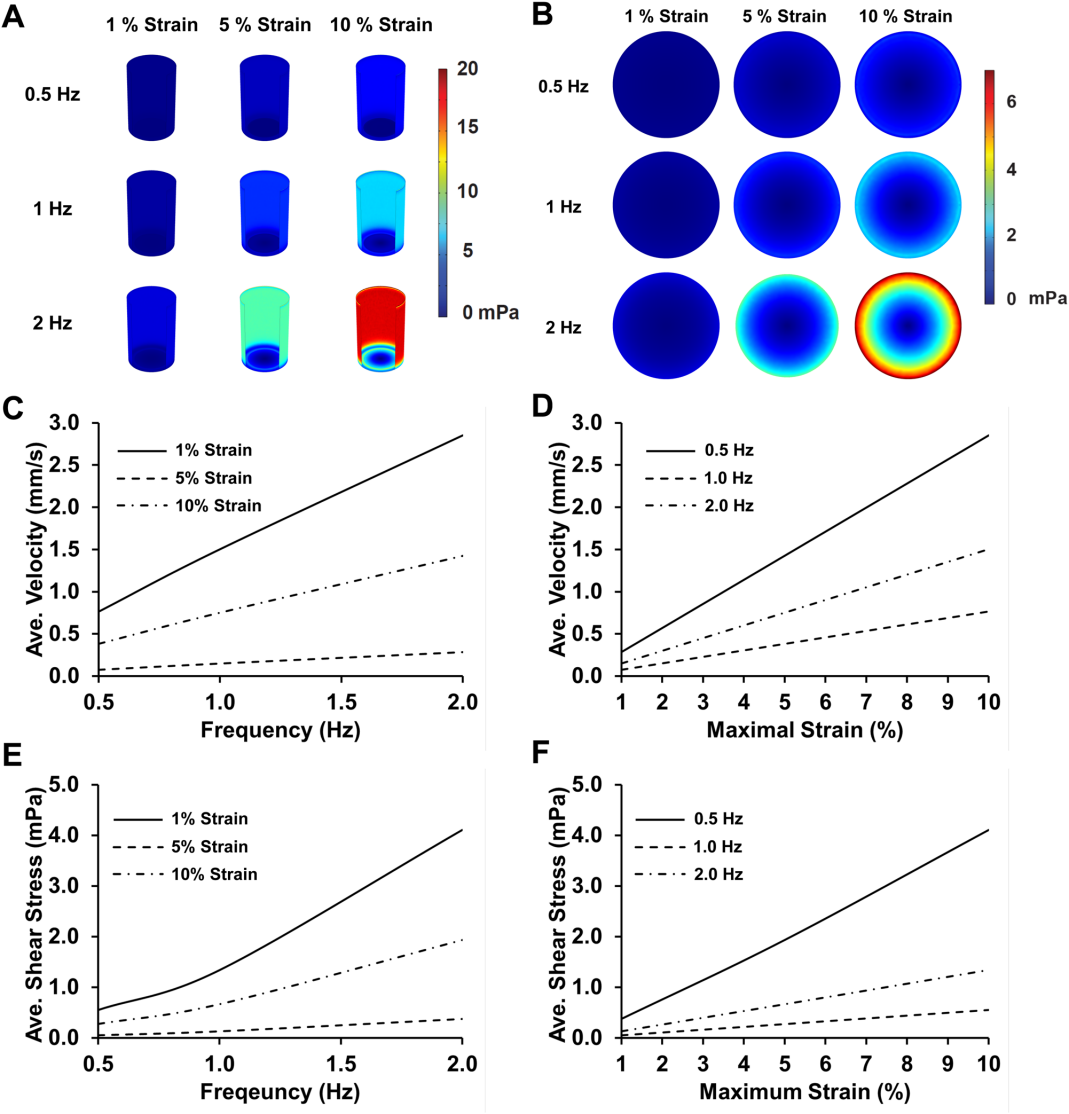
Computational modeling of fluid flow during mechanical loading. (**A**) Peak shear stress within the well during the displacement of the membrane. (**B**) Peak shear stress on the culture surface during mechanical loading. (**C**) Average fluid velocity within the well as a function of the frequency of loading. (**D**) Average fluid velocity within the well as a function of the maximal strain of loading. (**E**) Average shear stress on the culture surface as a function of the frequency of loading. (**F**) Average shear stress on the culture surface as a function of the maximal strain.

We created a high throughput mechanical loading system that allows the application of mechanical stretch to cells cultured in six 96-well plates simultaneously. The system applies mechanical load by displacing an array of pistons mounted on a platen through a flexible bottom culture plate. The high throughput biaxial oscillatory strain system (HT-BOSS) drives the motion of a platen using a tubular linear motor (Fig. 1A). Teflon pistons are mounted on the platen that can be driven to displace a flexible culture surface within a custom culture plate (Fig. 1B). This system can apply strains based on the displacement of the piston and there is a linear relation of displacement to strain for a broad range of mechanical strains (Fig. 2A,B). The height of each piston can be adjusted individually, allowing calibration of each piston for accurate strain application. We calibrated the strain applied to each of the 576 wells in the system and found a high degree of accuracy and repeatability for the strain application across all of the wells (Fig. 2C). The system uses a true linear (voice coil) motor that allows customizable displacements to create complex dynamic strain waveforms. We verified that the system could apply the sine waveform and two physiologic strain waveforms derived from the displacement of the arterial wall in the aorta and brachial arteries (Fig. 2D)^39^. As the motion of fluid within the cultured wells due to piston motion could create shear stress, we created a computational fluid flow model for the inside of the well during the application of mechanical strain to gauge the significance of this potential effect. From the simulations, we found that the average shear stress on the culture surface scaled in an approximately linear manner with the frequency of loading and maximum strain (Fig. 3). The shear stress applied was very low in magnitude and in the range of ~ 0.5 to 4 mPa over all of the conditions tested. In comparison to simulations of a larger format system with 35 mm diameter wells, the shear stress generated in this high throughput system was about tenfold lower (compared to 6.35 mm diameter wells)^39,40^. In the high throughput format, the system can apply a maximal of 17.5% strain in all of the wells.

### Multi-strain screening reveals that high level of mechanical strain leads to increased expression of Oct-4, Sox2 and SSEA1

**Figure 4:**
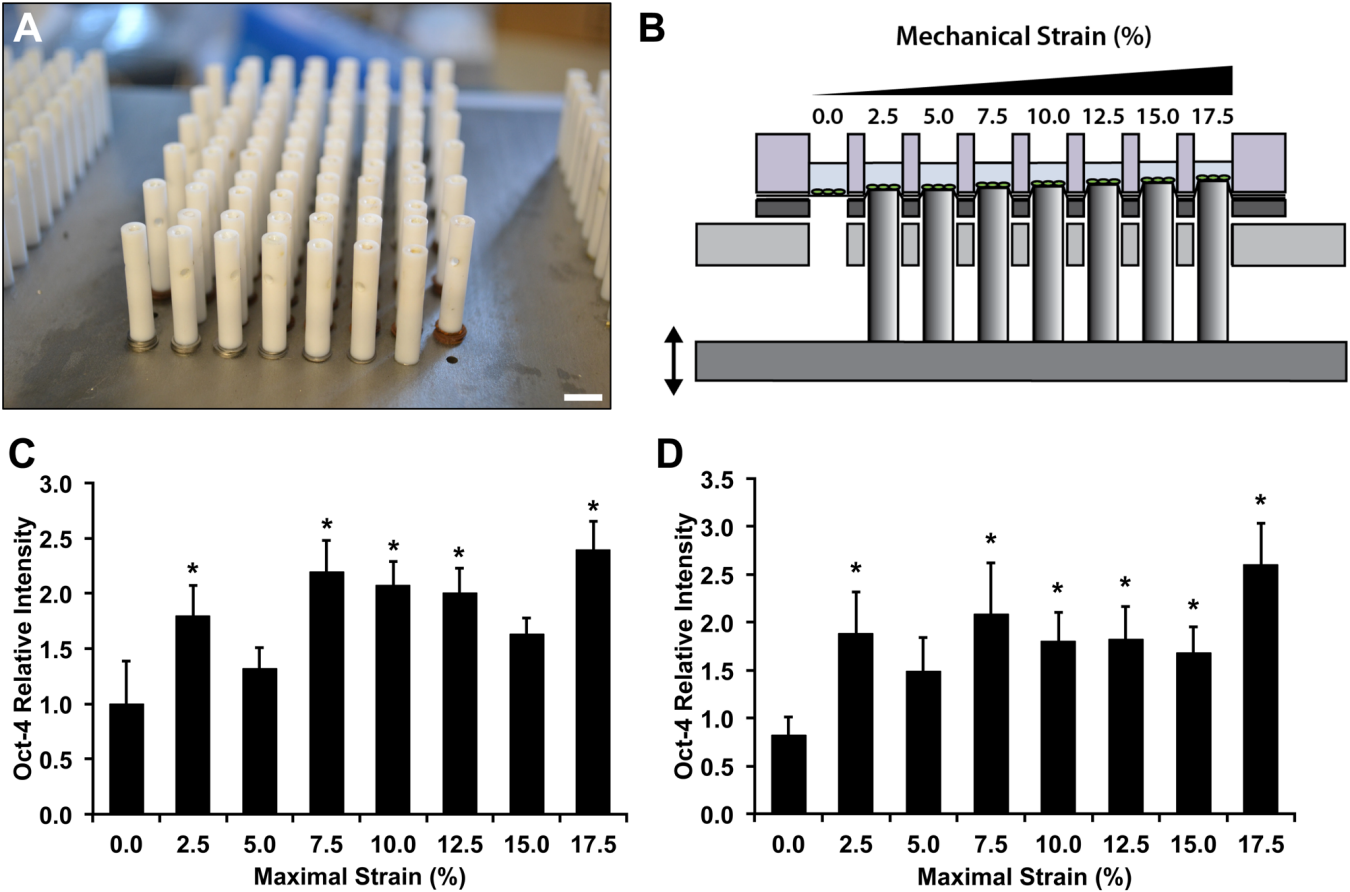
Mechanical strain enhances pluripotency transcription factors in mouse fibroblasts. (**A**) Thin shims were added to adjust the height of the pistons to apply varying mechanical strain across the plate. Bar = 1 cm. (**B**) Diagram of one row of pistons displacing the membranes. When the lowest piston is applying 2.5% strain the highest piston is applying 17.5% strain. Mechanical stain was applied at 0.1 Hz for four hours per day for seven days. (**C**) Expression of Oct-4 eGFP in MEFs after one day of mechanical loading at varying levels of strain. *p < 0.05 versus static control group. (**D**) Oct-4 eGFP signaling in MEFs after seven days of mechanical load. *p < 0.05 versus static control group.

**Figure 5:**
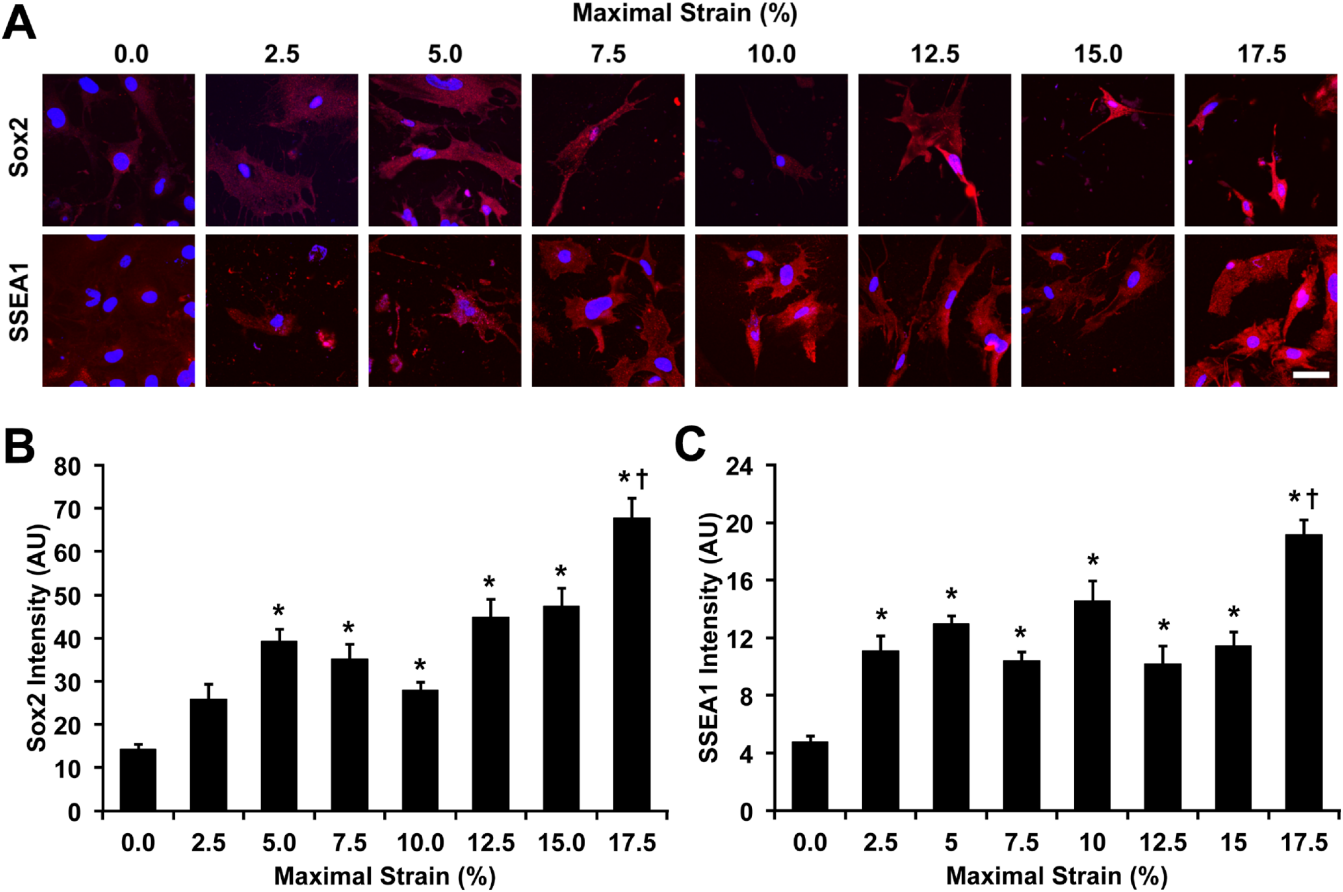
Mechanical strain increases expression of pluripotency factors Sox2 and SSEA1 in mouse fibroblasts. (**A**) Immunostaining for Sox2 and SSEA1 in MEFs treated with mechanical stain for 7 days. Bar = 50 µm. (**B**) Quantification of Sox2 in MEFs after 7 days of mechanical load. *p < 0.05 versus static control group. (**C**) Quantification of SSEA1 in MEFs after 7 days of mechanical load. *p < 0.05 versus static control group.

To test the ability of the system to perform mechanobiological screens, we examined the hypothesis that the expression of reprogramming factors may be regulated through the application of mechanical forces. Octamer-binding transcription factor 4 (Oct-4) is a transcription factor that is key in the maintenance and reinitiation of pluripotency^41^. We applied mechanical strain to mouse embryonic fibroblasts (MEFs) that expressed an Oct-4 enhanced green fluorescent protein (eGFP) transgene at 0.1 Hz with varying maximal strain from 0 to 17.5% strain. Using the high throughput loading system, we applied a range of load simultaneously by adjusting the heights of the pistons and calibrating the maximum strain applied (Fig. 4A,B). Using a plate reader, we assayed the expression of Oct-4 over seven days of loading (four hours of loading per day). We found that most levels of mechanical strain increased Oct-4 expression from approximately 1.5 to 2.5 fold and that the highest levels of Oct-4 were observed in cells exposed to 17.5% strain (Fig. 4C,D). Sox2 is a key transcription factor in the maintenance and development of pluripotency^42,43^. Stage specific embryonic antigen 1 (SSEA1) is an early marker of the development of pluripotency during the development of induced pluripotent stem cells from mouse embryonic fibroblasts (MEFs)^44^. We performed loading of MEFs under similar conditions and then immunostained for pluripotency factors Sox2 and SSEA1. We found significant increases in Sox2 at maximal strains greater than 5%, with a maximal increase at 17.5% strain (Fig. 5A,B). In addition, we found significant increases in SSEA1 with all levels of strain and a maximal increase with 17.5% strain (Fig. 5A,C).

### High throughput mechanobiological screen of small molecule inhibitors combined with mechanical load to enhance Oct-4 expression in MEFs

**Figure 6:**
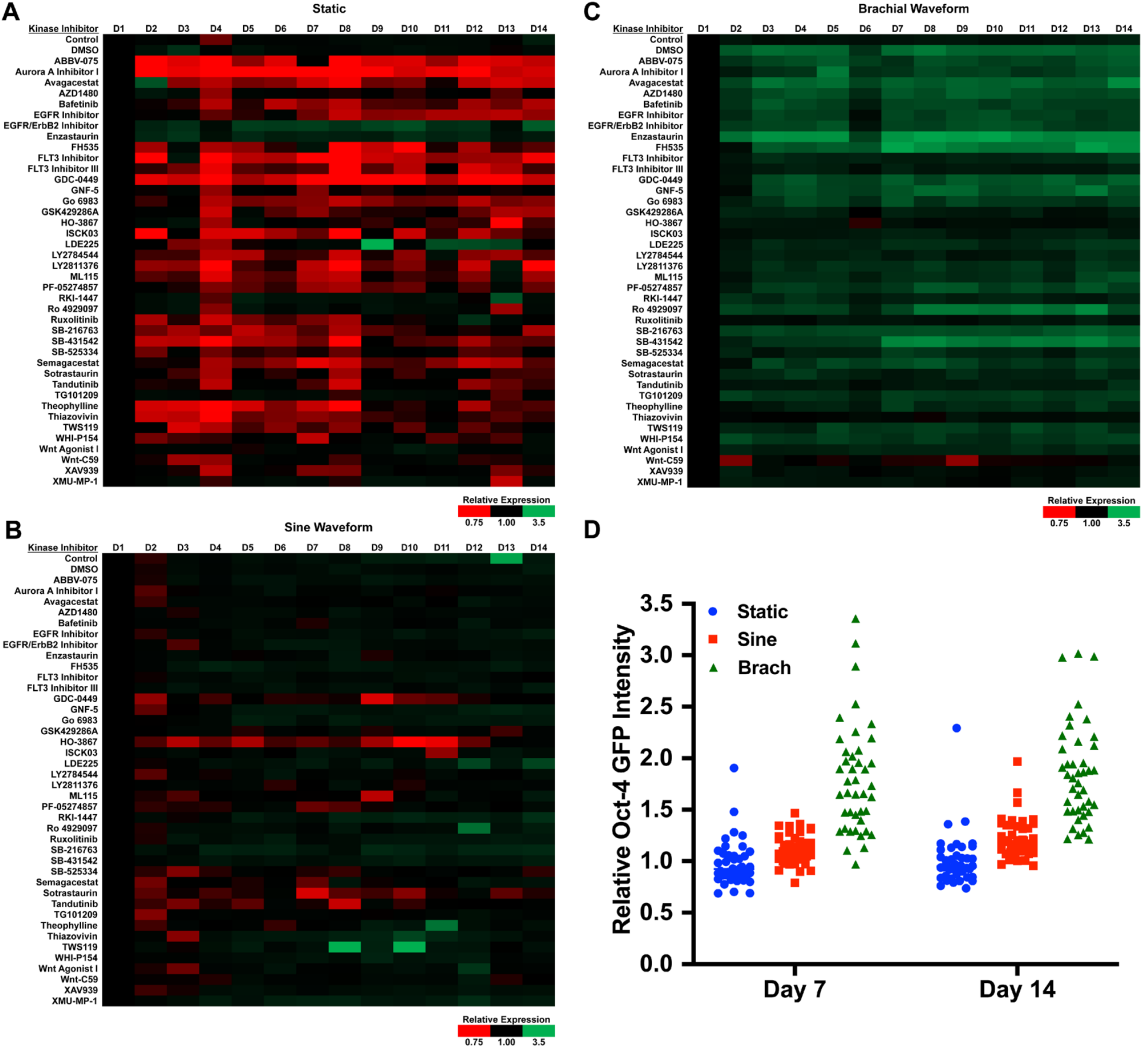
High throughput mechanobiological screen for small molecule inhibitors that synergistically increase Oct-4 GFP with mechanical loading. The MEFs were treated with 17.5% mechanical strain at 0.1 Hz for four hours per day for 14 days in the presence of compounds from a library of kinase inhibitors. The expression of Oct-4 GFP was measured using a plate reader each day. Heat maps of Oct-4 GFP fluorescence for MEFs under (**A**) static, (**B**) sine waveform or (**C**) brachial waveform loading. (**D**) Mean values of Oct-4 GFP expression after seven and fourteen days for all inhibitor treatments.

**Figure 7:**
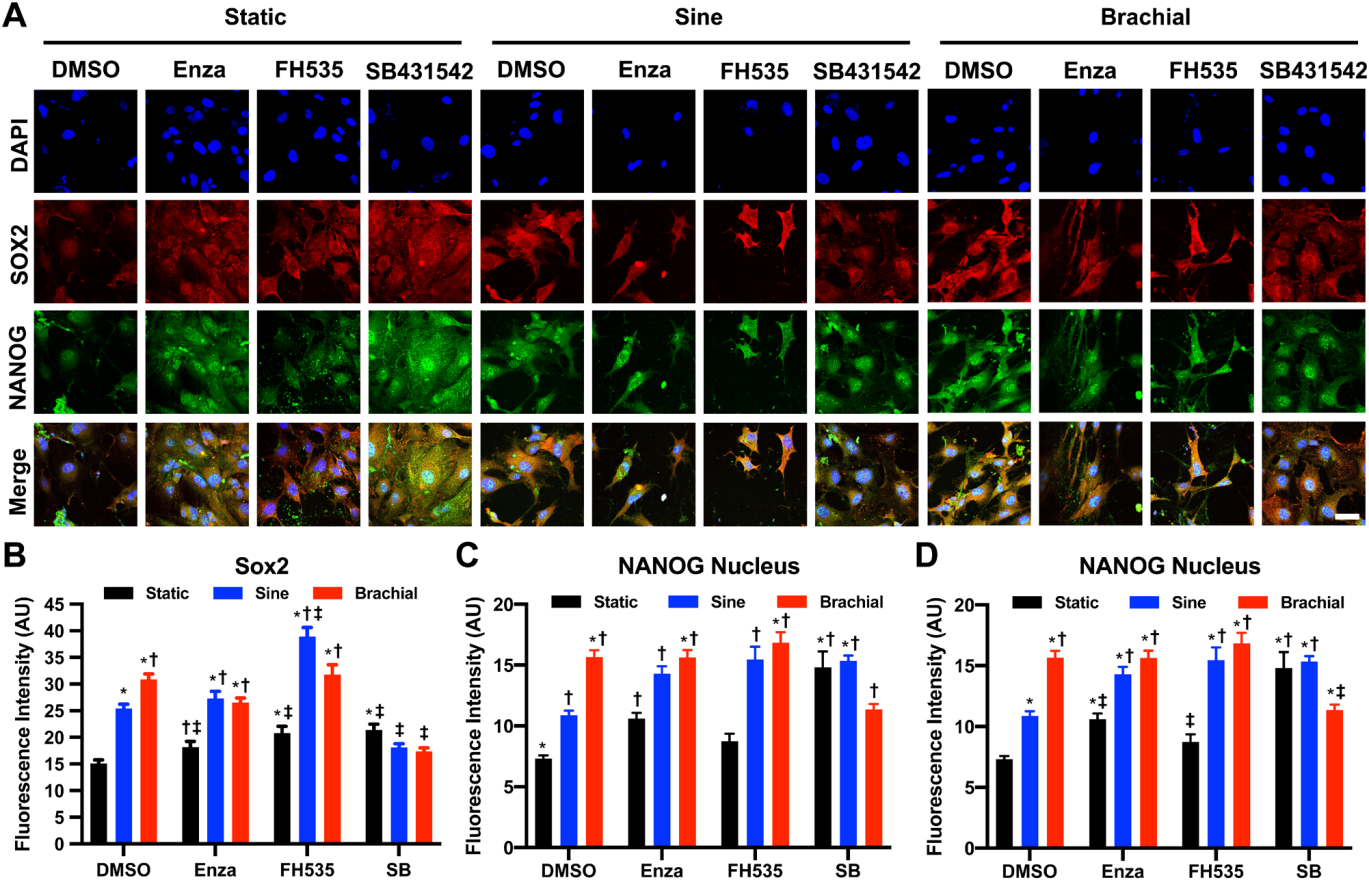
Optimized mechanical and pharmacological conditioning increases the expression of pluripotency markers in MEFs. (**A**) Immunostaining for Sox2 and NANOG in MEFs loaded under static, sinusoidal, or brachial waveform at 0.1 Hz, 17.5% maximal strain for 4 h a day for 14 days. Bar = 100 µm. (**B**) Quantification of Sox2 expression in MEFs after 14 days of mechanical load. *p < 0.05 versus static DMSO group. ^†^*p* < 0.05 versus sine DMSO group. ^‡^*p* < 0.05 versus brachial DMSO group. (**C**) Quantification of NANOG expression in the cytoplasm of the cells after 14 days of treatment. *p < 0.05 versus static DMSO group. ^†^*p* < 0.05 versus sine DMSO group. ^‡^*p* < 0.05 versus brachial DMSO group. (**D**) Measurement of NANOG expression in the nucleus of MEFs after 14 days of treatment. *p < 0.05 versus static DMSO group. ^†^*p* < 0.05 versus sine DMSO group. ^‡^*p* < 0.05 versus brachial DMSO group.

A key capability of the HT-BOSS system is the ability to perform drug screening in the presence of applied mechanical forces. This enables the investigation of synergy between mechanical forces and pharmacological agents. After observing the increases in reprogramming factors in MEFs, we then screen a small drug library to search for compound that may synergistically enhance reprogramming factor expression in MEFs. We performed a mechanobiological screen in which we applied 17.5% strain to Oct-4 eGFP MEFs at 0.1 Hz for 14 days. We applied the mechanical load using either a sinusoidal waveform or a brachial waveform that has a shape that mimics the strain in the brachial artery during the cardiac cycle. In addition, we treated the cells with a library of small molecule kinase inhibitors. Under pharmacological treatment in static conditions, many of the kinase inhibitors decreased Oct-4 expression below baseline levels in the MEFs (Fig. 6). In particular, inhibitors to the rho-associated protein kinase (ROCK), Ras homolog family member A (RhoA), Janus kinase (JAK) family receptors, signal transducer and activator of transcription-3 (Stat-3), c-kit and macrophage-stimulating protein-1/2 (MST1/2) inhibited the load-induced increase in Oct-4 expression. Cells treated with sine waveform and kinase inhibitors had predominantly either no alteration in Oct-4 or a reduction in Oct-4 expression (Fig. 6A–C). Under brachial waveform loading, there was increase in Oct-4 expression with most treatments (Fig. 6D). Notably, treatment with DMSO increased the Oct-4 expression in combination with brachial waveform mechanical loading in comparison to control cells with brachial loading. In addition, several of the kinase inhibitors increased the Oct-4 expression including a protein kinase Cβ (PKCβ) inhibitor (Enzastaurin; CAS 170364-57-5), a β-Catenin/Transcription Factor (Tcf) Inhibitor (FH535; CAS 108409-83-2), and a Glycogen Synthase Kinase-3 Kinase (GSK-3K) inhibitor (SB-431542; CAS 280744-09-4), in combination with brachial loading. To further investigate the expression of pluripotency related markers in the MEFs, we treated MEFs with a subset of conditions from our high throughput screen that increased Oct-4 GFP expression. NANOG is transcription factor that is important in maintaining pluripotency and reaching a pluripotent ground state for cells^45^. We then examined expression of Sox2 and NANOG by immunostaining. We found significant increases in Sox2 and NANOG with the brachial waveform and treatment with some of kinase inhibitors (Fig. 7). However, the inhibitors did not have synergistic increases in expression of Sox2 and NANOG in combination with brachial loading as seen with Oct-4.

## Discussion

While iPSCs have immense potential for their ability to mimic disease in vitro, there remain concerns for the use of genetically modified cells as therapeutics in patients. Thus, reprogramming strategies that do not use genetic modification would be highly advantageous for enhancing the therapeutic potential of iPSCs. Several studies have found that small molecule inhibitors can substitute for one or more pluripotency transcription factors^5–8^. However, prior studies have found that mechanical forces can both inhibit and enhance the development of pluripotency. In this study, we developed a novel mechanical screening system that enabled a more complete exploration of mechanical conditions that could potentially alter the development of pluripotency in somatic cells. Using this screening system, we found optimal mechanical conditions that enhanced the expression of pluripotency transcriptions factors in fibroblasts and that this could be further enhanced for Oct-4 through treatment with small molecule inhibitors.

In our study, high levels of mechanical strain were most effective in inducing pluripotency transcription factor expression. In addition, the use of a physiologic waveform (brachial) with a high strain rate was optimal for inducing Oct-4 expression in fibroblasts. The expression of Oct-4 in MEFs was further increased by adding in inhibitors to PKCβ, β-catenin/Tcf, or TGF-βRI/ALK4/ALK7 in combination with the optimal mechanical loading conditions. Mechanical strain alone was able to increase Oct-4 eGFP expression by nearly 2.5 fold. This effect was present after one day of loading and increased slightly over time for the seven days. With the addition of specific inhibitors with mechanical load, the enhancement of Oct-4 eGFP was increased to 3.2 fold over baseline levels. In other reprogramming factors, brachial loading did increase the expression of Sox2 and NANOG. However, the inhibitors identified from the Oct-4 screen did not further increase the effect with brachial waveform loading on these reprogramming factor and in some cases blocked the increase caused by the mechanical loading. These findings suggest that the small molecules identified in the screen are specific to Oct-4 in their ability to enhance the load-induced effects on reprograming factor expression.

One potential limitation of our studies is the use of immunofluorescent assays for the assessment of the pluripotent markers. While this technique has limitations, it interfaces well with standard high content cell screening methods and allows the quantification of cell response on the single cell level, allowing an assessment of cell-to-cell variability, subpopulation response and heterogeneity (common issues in studies using stem cells). Genetic reporter constructs such as the Oct-4 reporter used in this study can overcome some of these limitations but also have caveats for use in human cells where viral transduction and selection with antibiotics are required for establishing stable cell lines. In this study, we used cells derived from a transgenic mouse, which did not require viral transduction.

Overall, our results demonstrate the ability of combined mechanical/pharmacological conditioning to prime the pluripotency of somatic cells in the absence of genetic modification. The induction of the transcription factors was highly dependent on the magnitude of mechanical strain and the complex dynamics of the applied waveform. Moreover, the response of the cells to drugs was highly dependent on the mechanical conditions, with compounds causing opposing response in static versus loaded conditions. Thus, mechanobiological screening may provide complementary enhancement of strategies for chemically inducing pluripotency and aid in the development of safe methods for creating iPSCs for therapy in human patients.

## Materials and methods

### Cell culture

Mouse embryonic fibroblasts (PrimCells LLC) or Oct-4 eGFP reporter expressing MEFs (EMD Millipore) at passages 5 were cultured in 4.5 g/L d-glucose DMEM medium supplemented with 10% fetal bovine serum, l-glutamine, and penicillin/streptomycin. The MEFs were obtained from CF1 mice and were derived from 13-day-old embryos (pooled cells from male and female mice). All cells were cultured at 37 °C and 5% CO_2_. For passaging, 70% confluent MEFs were first washed with PBS. The cells were agitated with 0.25% EDTA-trypsin at 37 °C for 1 min. Cell suspension was then collected and centrifuged at 500*g* for 5 min. The cells were then seeded to a new substrate at 2,000 cells per cm^2^.

### Mechanical loading device

Strain was applied to cells using a custom made device that displaces pistons through the flexible culture surface. The device operates using a true linear motor that drives a platen on motion rails. Linear ball bearings are used along the rails to minimize friction, while fixed springs on the rods help reduce the load on the motor and prevent the platen from moving while the device is turned off. There are 576 individual polytetrafluoroethylene (PTFE) pistons mounted on the platen that are removable and can be calibrated individually through thin shims. A top plate on the system has mounting holes for custom designed culture plates that hold a silicone membrane sandwiched between steel plates and silicone gaskets. The silicone membrane can be coated to allow culture of cells and the entire geometry matches that of a standard 96 well culture plate. Mechanical strain is applied when the pistons are moved into the silicone membrane, causing displacement and application of stretch to the cells. The linear motor is hygienically sealed and feedback controlled by software that regulates the current through the coils around the motor (Copley Motion). The motor has a resolution of displacement of 10 μm. To avoid excess heat from the current generated and from high temperature inside cell incubators, a cooling system is integrated with an external water bath circulating chilled water into the motor’s enclosure.

### Flexible-bottom culture plate assembly

The cell culture plate was assembled by sandwiching custom made parts with the flexible silicone rubber to provide a cell culture substrate. The culture plate consists of a polycarbonate top plate and aluminum bottom plate that are held together by eight screws. Sandwiched between the top and bottom plate is a 0.005′′ thick silicone sheet (Specialty Manufacturing, Inc.) with a rubber gasket to prevent leaking. The plates are sterilized prior to cell seeding by UV light and are coated by treatment with 50 µg/mL fibronectin at 37 °C overnight. On the day of mechanical loading, the plates were mounted to the top plate of the device with eight additional screws.

### Calibration of mechanical strain

Mechanical strain applied to the flexible membrane was measured by recording changes in the marks drawn on the membrane. A uniform array of marks was created on the cell culture surface using silicone glue. The pistons were displaced at small increments through the membrane and a high magnification image of the mark was recorded using a high-resolution camera (Basler AG). The displacement of the membrane was measured using Metamorph Imaging software (version 7.8; Molecular Devices, Sunnyvale, CA). For dynamic mechanical loading, strain was measured by recording a video at 60 frames per second. The membrane was displaced in sinusoidal, aortic, and brachial waveforms created from clinical arterial distension data^39^. For each waveform, three cycles of the waveform were recorded and averaged.

### Computational modeling of the system

Fluid mechanics in the cell culture media was modelled using finite element software (COMSOL). Briefly, a cylindrical shape fluid structure was used to model a single well with viscosity and density of standard Dulbecco's modified Eagle's medium (DMEM) media. The bottom surface was displaced in a sinusoidal motion over time with three frequencies (0.1 Hz, 1 Hz, and 2 Hz) in combination with three maximum magnitudes (1%, 5%, and 10%). A series of mesh and tolerance optimization were performed to optimize these parameters. The maximum and average fluid velocities at various cross sections were computed. Average shear stress in various locations and over various time points were computed as well with the focus on the location of the bottom surface undergoing displacement where the cells are located.

### Immunostaining

Following the treatments, the cells were fixed in 4% paraformaldehyde in PBS for 10 min followed by washing and permeabilization with 0.1% Triton X-100 PBS for 5 min. Next, samples were blocked with PBS containing 5% fetal bovine serum (FBS) and 1% bovine serum albumin (BSA) for 40 min. After washing, cells were incubated with primary antibodies at 1:100 dilution ratio in PBS with 1% BSA overnight at 4 °C. Primary antibodies used include NANOG (ab80892; Abcam), Sox2 (4744S; Cell Signaling Technology), and SSEA-1 (ab79351; Abcam). The samples were then washed twice in phosphate buffered saline (PBS) with 1% BSA and incubated with secondary antibodies at 1:1,000 dilution ratio in PBS with 1% BSA for 2 h in a light protected environment. Cells were then washed with PBS with 1% BSA prior to mounting in anti-fade media (Vector Laboratories, Inc.). The samples were imaged using FV10i Confocal Laser Scanning Microscope (Olympus, Inc.). Images of the fluorescent cell cultures were then traced in Adobe Photoshop for fluorescence intensity quantification.

### Multi-strain mechanical loading and measurement of Oct-4 GFP expression

To determine the optimal strain for Oct-4 expression in MEF, Oct-4 eGFP reporter expressing MEFs were exposed to either static or sinusoidal waveform loading for 8 h a day for 7 days using the multi-strain configuration of HT-BOSS, where the cells were exposed to maximal strain ranging from 0.0 to 17.5%, in 2.5% intervals. For measuring the expression of Oct-4 eGFP, plate reader was used (Varioskan; Thermo Fisher). Briefly, culture media was replaced with Tyrode’s Solution every day prior to the fluorescence measurement. After plate reading, the Tyrode’s solution was replaced with cell culture media.

### Kinase inhibitor drug library

For the kinase screening study, Oct-4 eGFP expressing MEFs were cultured under static, sinusoidal waveform, or brachial waveform loading for 4 h per day for 14 days at 0.1 Hz and 17.5% maximal strain. During loading, the cells were treated with media no treatment, 1 μM DMSO, or one of the 40 kinase inhibitors listed in Supplemental Table 1 at 1 μM (Cayman Chemicals). Each day, the cell culture media were replaced with Tyrode’s Solution and the Oct-4 GFP expression was measured using a plate reader. The Tyrode’s solution was replaced with media containing treatment every day prior to stretching.

### Statistical analysis

All results are shown as mean ± standard error of the mean. Multiple comparisons between groups were analyzed by two-way ANOVA followed by a Tukey post-hoc or a Dunnett post-hoc test when testing multiple comparisons versus a control group. A *p*-value of 0.05 or less was considered statistically significant.

## Supplementary information

Supplementary Table.
