# Supplementary material for: A high throughput screening system for studying the effects of applied mechanical forces on reprogramming factor expression
Source: Sci Rep. 2020 Sep 22;10:15469. doi: 10.1038/s41598-020-72158-5 (PMC7508814; doi:10.1038/s41598-020-72158-5)
Supplement: Supplementary file 1 — Supplementary Table. [file 41598_2020_72158_MOESM1_ESM.docx]

**Supplemental Tables**

| **Supplemental Table 1.** *Kinase Inhibitors Used in the Studies* | | |
| --- | --- | --- |
| **Kinase Inhibitor Target** | **Catalog Number** | **CAS Number** |
| ABBV-075 | 21033 | 1445993-26-9 |
| Aurora A Inhibitor I | 21600 | 1158838-45-9 |
| Avagacestat | 16711 | 1146699-66-2 |
| AZD1480 | 10702 | 935666-88-9 |
| Bafetinib | 19169 | 859212-16-1 |
| EGFR Inhibitor | 15363 | 879127-07-8 |
| EGFR/ErbB2 Inhibitor | 17568 | 179248-61-4 |
| Enzastaurin | 11601 | 170364-57-5 |
| FH535 | 18001 | 108409-83-2 |
| FLT3 Inhibitor | 18462 | 301305-73-7 |
| FLT3 Inhibitor III | 21193 | 852045-46-6 |
| GDC-0449 | 13613 | 879085-55-9 |
| GNF-5 | 16254 | 778277-15-9 |
| Go 6983 | 13311 | 133053-19-7 |
| GSK429286A | 15262 | 864082-47-3 |
| HO-3867 | 21581 | 1172133-28-6 |
| ISCK03 | 15781 | 945526-43-2 |
| LDE225 (phosphate) | 16263 | 1218778-77-8 |
| LY2784544 | 16705 | 1229236-86-5 |
| LY2811376 | 16712 | 1194044-20-6 |
| ML115 | 15178 | 912798-42-6 |
| PF-05274857 (HCl) | 15022 | 1613439-62-5 |
| RKI-1447 | 16278 | 1342278-01-6 |
| Ro 4929097 | 19996 | 847924-91-1 |
| Ruxolitinib | 11609 | 941678-49-5 |
| SB-216763 | 10010246 | 280744-09-4 |
| SB-431542 | 13031 | 301836-41-9 |
| SB-525334 | 16281 | 356559-20-1 |
| Semagacestat | 16713 | 425386-60-3 |
| Sotrastaurin | 16726 | 425637-18-9 |
| Tandutinib | 12098 | 387867-13-2 |
| TG101209 | 14696 | 936091-14-4 |
| Theophylline | 23760 | 58-55-9 |
| Thiazovivin | 14245 | 1226056-71-8 |
| TWS119 | 10011251 | 601514-19-6 |
| WHI-P154 | 16178 | 211555-04-3 |
| Wnt Agonist I | 19903 | 853220-52-7 |
| Wnt-C59 | 16644 | 1243243-89-1 |
| XAV939 | 13596 | 284028-89-3 |
| XMU-MP-1 | 22083 | 2061980-01-4 |
